# Remote Monitoring of Implantable Cardioverter Defibrillator

**Published:** 2006-10-01

**Authors:** Dwight W Reynolds, N Jayaprasad, Johnson Francis

**Affiliations:** 1 Cardiovascular Section, The University of Oklahoma Health Sciences Center, Oklahoma City, Oklahoma, USA; 2 Department of Cardiology, Calicut Medical College, Kerala, India

**Keywords:** Implantable cardioverter defibrillator, telemonitoring, internet based monitoring

## Introduction

The rate of implantable cardioverter defibrillator (ICD) implantation has gone up as primary and secondary prevention trials have relatively consistently shown significant improvement in mortality and morbidity. Most patients with ICDs are followed routinely at intervals ranging from 3 to 6 months. Many patients require additional non-scheduled visits to investigate symptoms that may or may not relate to their cardiac disease or device. Appropriate and inappropriate therapies of implantable cardioverter defibrillators have a major impact on morbidity and quality of life in ICD recipients. Remote monitoring systems can substitute for routine follow-up visits and/ or deliver continuous diagnostic and device status information. Remote monitoring of ICDs can decrease the need for many patient visits and, thereby, probably reduce expense.

## Remote Monitoring

In the early 1970s, the concept of trans telephonic monitoring (TTM) was introduced to monitor the longevity of pacemakers [1]. This modality was identified as a useful method for tracking the basic function of pacemaker systems; particularly in the early era of pacemaker development when both lead and pulse generator longevities were unpredictable. In the late 1970s and 1980s, the usefulness of TTM as a diagnostic tool expanded to other problems including sensing, capture, lead malfunction, and arrhythmias. As the sophistication of pacemakers increased,  the modes of follow up also inevitably changed. Most pacemaker manufacturers now have systems in place that facilitate remote pacemaker follow-up. Transmissions can be made totally trans-telephonically or, more recently, by using web-based networks. Implanted devices can be interrogated by using easy-to-use equipment in patients' homes (or elsewhere) that communicates stored and real-time information trans-telephonically to a secure server where the data are available to the pacemaker/heart rhythm professional.

Just as for pacemakers, there are currently several systems either available or in development for the remote interrogation and monitoring of ICDs. Each system can only be used with devices manufactured by the same company. *Cardiomessenger & Home Monitoring* from Biotronik uses diagnostic data transmitted from pacemakers, ICDs and heart failure devices to a mobile phone-like unit, then, in turn via cellular phone network, to a secure, remote monitoring center, and then further forwarded on to patients' clinical teams. Data is generated and transmitted at set times, following an event, or with patient-triggering. Similar in concept, *CareLink* from Medtronic, *Housecall Plus* from St. Jude Medical, and *Latitude* from Boston Scientific/Guidant are remote monitoring systems that use standard telephone lines to communicate with clinicians and/or secure computer servers which can be accessed by clinicians. Depending on the system, comprehensive device information including electrograms can be collected from the implanted device and transmitted and stored for clinician access and use.

## Usefulness

By diminishing the frequency of clinic visits, remote monitoring may reduce health care costs and patients' inconvenience. Diagnostic data from devices such as the numbers of aborted and delivered ICD therapies are an indicator of the total incidence of tachyarrhythmia. Frequently recurring episodes of ventricular tachyarrhythmias in patients may indicate increasing instability and progression of cardiac disease [2]. Inappropriate ICD therapy, typically related to sinus tachycardia or other superventricular tachycardia can be elucidated remotely as well as in clinic [3].  In one study, 59 patients completed 119 transmissions. Clinician review of data transmissions revealed several clinically significant findings, including silent AF discovery, assessment of antiarrhythmic drug efficacy in a previously diagnosed AF patient, previously unobserved atrial undersensing, and ventricular tachycardia. Clinicians were satisfied with the performance of the network and the quality of the web-accessed data, and found it comparable to an in-office device interrogation [4].  In another prospective evaluation, 124 patients with single chamber ICDs were monitored by remote interrogation and monitoring for a total of 570 transmissions.  The investigators demonstrated a high degree of patient satisfaction with the convenience and ease of use of remote ICD monitoring [5].   Intracardiac electrograms (IEGMs) stored in the ICD can be essential for differentiating appropriate and inappropriate ICD therapies. High quality IEGMs are increasingly available with implanted devices and the remote monitoring systems.  Validation of adequacy of remotely acquired IEGMs is under study [6]

Remote monitoring can be especially useful for monitoring patients with CRT-D devices. A prospective, longitudinal, multicenter home monitoring trial was conducted in 123 patients with clinical indications for CRT. In a mean follow-up period of 3 months, 11 unplanned re-hospitalizations for cardiovascular causes and 9 deaths occurred.  In 70% of the re-hospitalization events, the retrospective analysis of remotely acquired data revealed an increase in mean heart rate at rest and in mean heart rate over 24 h within 7 days preceding hospitalization. These interim findings suggest that remotely acquired data may predict events leading to hospitalization and encourage further research [7]. Other implantable devices using transthoracic impedance sensing in ICDs, and RV pressure parameter monitoring have been used with remote monitoring to assist in caring for patients with heart failure.

An important and untapped potential utility for remotely monitored devices in which data is collected in large databases involves the capacity to predict device performance problems.

## Cost Effectiveness

In a cost effectiveness study conducted among 502 patients, costs of conventional follow-up of ICDs were calculated without and compared with the expected cost of follow up with remote monitoring. It was concluded that the remote monitoring may considerably reduce the overall costs of ICD follow up by saving on transportation cost, particularly when the distance between home and medical facility is >100 km [8]. Patient satisfaction with the convenience and reliability of the remote monitoring system ranged from 93 to 97% in SF-36 surveys [9]

## Conclusion

Remote interrogation provides frequent, convenient, safe and comprehensive monitoring of ICDs. Device and patient related problems can be reliably detected and reduce the frequency of outpatient visits. Patients are highly satisfied with the convenience and ease of use of the systems. Surveillance of device performance is a potentially important future capability. In the future an exponential growth in implementation of remote monitoring is likely to occur in the field of implanted devices including ICDs, pacemakers, and implantable disease monitors.
